# Costunolide Plays an Anti-Neuroinflammation Role in Lipopolysaccharide-Induced BV2 Microglial Activation by Targeting Cyclin-Dependent Kinase 2

**DOI:** 10.3390/molecules25122840

**Published:** 2020-06-19

**Authors:** Yan-Chen Liu, Na Feng, Wei-Wei Li, Peng-Fei Tu, Jian-Ping Chen, Jing-Yan Han, Ke-Wu Zeng

**Affiliations:** 1 Department of Integration of Chinese and Western Medicine, School of Basic Medical Sciences, Peking University, Beijing 100191, China; liuyanchen@bjmu.edu.cn; 2 State Key Laboratory of Natural and Biomimetic Drugs, School of Pharmaceutical Sciences, Peking University, Beijing 100191, China; 1816391006@bjmu.edu.cn (N.F.); pengfeitu@bjmu.edu.cn (P.-F.T.); 3 Integrated Laboratory of Chinese and Western Medicine, Peking University First Hospital, Beijing 100034, China; liweiwei@pkufh.com; 4 Tasly Microcirculation Research Center, Peking University Health Science Center, Beijing 100191, China; 5 School of Chinese Medicine, the University of Hong Kong, Hong Kong 999077, China; jianpingchen@hku.hk

**Keywords:** costunolide, natural product, anti-neuroinflammation, target identification, CDK2

## Abstract

Hyperactivation of microglia in the brain is closely related to neuroinflammation and leads to neuronal dysfunction. Costunolide (CTL) is a natural sesquiterpene lactone with wide pharmacological activities including anti-inflammation and antioxidation. In this study, we found that CTL significantly inhibited the production of inflammatory mediators including nitric oxide, IL-6, TNF-α, and PGE2 in lipopolysaccharide (LPS)-stimulated BV2 microglia. Moreover, CTL effectively attenuated IKKβ/NF-κB signaling pathway activation. To identify direct cellular target of CTL, we performed high-throughput reverse virtual screening assay using scPDB protein structure library, and found cyclin-dependent kinase 2 (CDK2) was the most specific binding protein for CTL. We further confirmed the binding ability of CTL with CDK2 using cellular thermal shift assay (CETSA) and drug affinity responsive target stability (DARTS) assays. Surface plasmon resonance analysis also supported that CTL specifically bound to CDK2 with a dissociation constant at micromole level. Furthermore, knocking down CDK2 obviously reversed the anti-inflammation effect of CTL via AKT/IKKβ/NF-κB signaling pathway on BV-2 cells. Collectively, these results indicate that CTL inhibits microglia-mediated neuroinflammation through directly targeting CDK2, and provide insights into the role of CDK2 as a promising anti-neuroinflammation therapeutic target.

## 1. Introduction

Neuroinflammation is a crucial pathological process of various neurodegenerative diseases [1]. Microglia are major immune cells in brain [2]. In physiological status, microglia are in a resting or task-negative state [3]. However, microglia can be over-activated under continuous noxious stimuli, resulting in inflammatory mediator release as well as further neuronal damage [4,5]. Due to a shortage of efficient drugs and high toxic side effects, novel anti-neuroinflammation agents need to be developed for clinical application.

Natural products derived from medicinal herbs play an important role in developing innovative medicines to treat inflammatory diseases [6]. Costunolide (CTL) is a principal active sesquiterpene lactone from medicinal plant *Aucklandia lappa* Decne [7]. CTL has been reported to exhibit various health benefits including anti-inflammatory [8], antioxidative [9], anticarcinogenic [10,11,12], and antiallergic properties [13]. Recently, the anti-inflammatory activity of CTL has especially attracted great attention. Investigations in vivo have demonstrated that CTL alleviates heat-killed *Staphylococcus aureus* or lipoteichoic acid-induced acute lung injury via inhibition of pulmonary neutrophil infiltration [14]. Moreover, CTL also ameliorates experimental pleurisy [15] and ethanol-induced gastric ulcer [16] in mice. However, the anti-inflammatory mechanism of CTL and its potential molecular targets are still unclear.

In this study, we confirmed that CTL significantly inhibited microglia-mediated neuroinflammation by suppressing several inflammatory mediators [17]. Moreover, we identified cyclin-dependent kinase 2 (CDK2) as a direct cellular target of CTL. CTL exerts anti-neuroinflammation via CDK2-dependent AKT/IKKβ/NF-κB signaling pathway. These findings indicate CTL is a potential candidate agent for anti-neuroinflammation, and CDK2 may act as a promising therapeutic target for treatment of neuroinflammation.

## 2. Results

### 2.1. CTL Inhibited LPS-Induced Neuroinflammation Response in BV2 Cells

**Figure 1:**
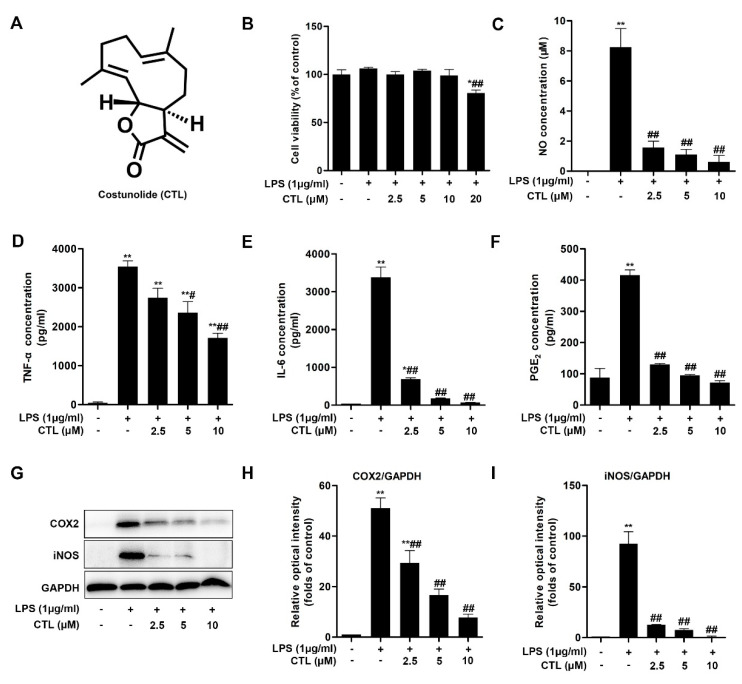
Costunolide inhibits lipopolysaccharide (LPS)-induced neuroinflammation response in BV2 cells. (**A**) Costunolide. (**B**–**G**) BV2 microglia were treated with gradient concentrations of Costunolide (CTL) and 1 μg/mL of LPS or vehicle for 24 h. (**B**) The cell viability was determined by MTT method. The levels of NO (**C**), TNF-α (**D**), IL-6 (**E**) and PGE_2_ (**F**) were detected by Griess method and ELISA assay. (**G**) The protein expression of COX2 and inducible nitric oxide synthase (iNOS) were detected by Western blot assay. Quantitative analysis for relative levels of cyclooxygenase2 (COX2) (**H**), and iNOS (**I**) were performed by normalizing to the control group. Data are mean ± SEM for three individual experiments. * *p* < 0.05, ** *p* < 0.01 vs. control group. ^#^ *p* < 0.05, ^##^ *p* < 0.01 vs. LPS group. *p* values were calculated by ANOVA with Bonferroni’s post hoc test.

We first tested the potential cytotoxicity of CTL on LPS-stimulated BV2 microglial cells by MTT assay, and found that CTL (2.5, 5, and 10 μM) [7] had no effect on cell survival, but CTL was cytotoxic at 20 μM (Figure 1B). Thus, the concentration range of CTL (2.5, 5, and 10 μM) was appropriate for analyzing its role in LPS-stimulated neuroinflammation. We found CTL significantly blocked LPS-induced NO release in the way of concentration-dependent (Figure 1C). Moreover, CTL inhibited LPS-induced various proinflammatory cytokines secretion such as TNF-α, IL-6 and PGE2 (Figure 1D–F). In addition, the role of CTL in inhibiting neuroinflammation was confirmed by significantly reduce expressions of cyclooxygenase2 (COX2) and inducible nitric oxide synthase (iNOS) (Figure 1G). The above results indicate that CTL is an effective inhibitor against microglia-mediated neuroinflammation.

### 2.2. CTL Effectively Attenuates LPS-Induced IKKβ/NF-κB Pathway Activation

**Figure 2:**
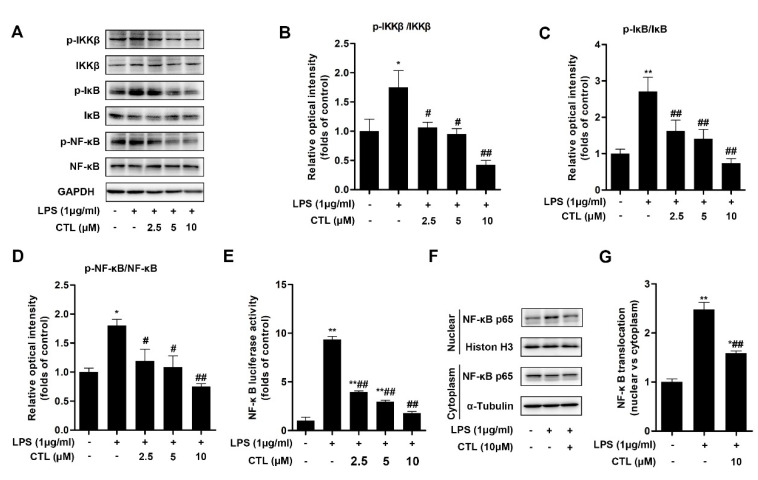
Costunolide attenuates LPS-Induced IKKβ/NF-κB Pathway Activation. (**A**) BV2 microglia were treated with gradient concentrations of CTL and 1 μg/mL of LPS or vehicle for 1 h. Phosphorylation and total expressions of IKKβ, IκB-α, and NF-κB p65 were determined by Western blot assay. (**B**–**D**) Quantitative analysis for relative phosphorylation levels of IKKβ (**B**), IκB-α (**C**), and NF-κB p65 (**D**). (**E**) BV2 microglia were transfected with NF-κB and Renilla reporter plasmids for 48-72 h, subsequently the cells were treated with gradient concentrations of CTL and 1 μg/mL of LPS or vehicle for 12 h and subjected to luciferase assay. CTL treatment notably deactivated NF-κB p65 subunit, as indicated by decreasing NF-κB transcriptional activity (**E**) and subsequently suppressing nuclear translocation (**F**). (**G**) Quantitative analysis for relative levels of nuclear vs. cytoplasm. Data are mean ± SEM for three individual experiments. * *p* < 0.05, ** *p* < 0.01 vs. control group. ^#^ *p* < 0.05, ^##^ *p* < 0.01 vs. LPS group. *p* values were calculated by ANOVA with Bonferroni’s post hoc test.

Canonical NF-κB pathway has been widely covered as a central feature in LPS-induced immunity and inflammatory responses [18]. Thereafter, we detected the activation of pivotal biomarkers of NF-κB pathway in BV2 microglial cells induced by LPS stimulation. Our results showed that, due to LPS stimulation, the phosphorylation levels of IKKβ and IκB-α were enhanced, at the same time IκB-α protein expression was significantly decreased. However, CTL treatment prominently down-regulated IKKβ and IκB-α phosphorylation, and blocked IκB-α degradation simultaneously (Figure 2A). We also used luciferase reporter gene assay to examine the effect of CTL on NF-κB transcriptional activity. As shown in Figure 2E, transcriptional activity of NF-κB in LPS group was significantly higher than that of the control group, which was inhibited in a concentration-dependent manner upon CTL treatment. In addition, we observed nuclear translocation of NF-κB p65 subunit and its augmented phosphorylation in LPS-induced BV2 microglial cells. CTL treatment can notably deactivate NF-κB p65 subunit, including suppressed its phosphorylation and nuclear translocation (Figure 2F). Collectively, these results indicated that CTL can inhibit IKKβ/NF-κB pathway activation against LPS stimulation, thereby exerting anti-neuroinflammation effect.

### 2.3. Cellular Target Identification of CTL

**Figure 3:**
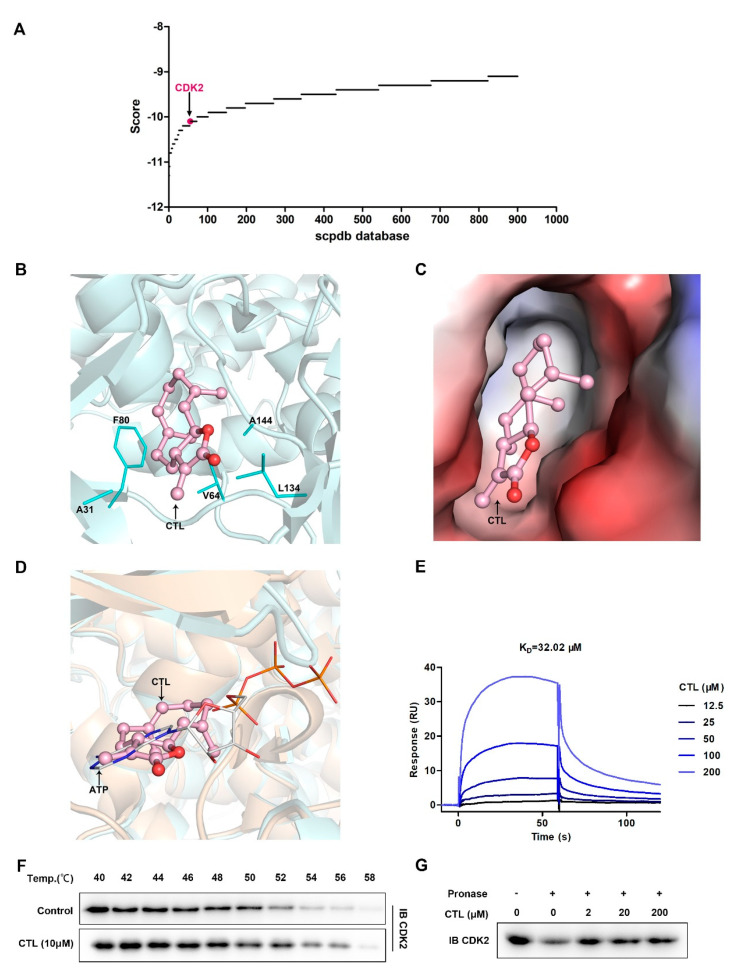
Cyclin-dependent kinase 2 (CDK2) is selectively targeted by CTL. (**A**) Target proteins identification by reverse virtual screening technology and CDK2 was identified as a crucial cellular target of CTL. (**B**) The binding site of CDK2 (only residues formed hydrophobic interaction with CTL are displayed) with CTL (pink in ball-and-stick representation) was shown as three-dimensional structure. (**C**) Electrostatic surfaces of CDK2 in complex with CTL. Negative charges are highlighted in red and positive charges in blue. (**D**) Superposition of structures showing CDK2-CTL and CDK2-ATP (PDBID: 4eoo), showing that CTL competitively occupied the ATP binding site with CDK2. CTL in ball-and-stick mode is colored in pink, ATP in line mode is colored in orange. (**E**) Surface plasmon resonance spectroscopy analysis graph suggests that concentration-dependent of CTL specifically bound to CDK2 with a dissociation constant at micromole level. The kinetic parameters of dissociation constant (KD) were derived by fitting to a 1:1 Langmuir binding model. (**F**) BV2 cells were exposed to CTL (10 μM) or vehicle followed by a cellular thermal shift assay. (**G**) BV2 cell lysates was incubated with CTL in the presence or absence of pronase (2 μg/mL). CTL concentration-dependently suppressed protease induced-degradation of CDK2 in drug affinity responsive target stability assay.

To elucidate the cellular target of CTL, we used receptor-based reverse virtual screening method. It is based on molecular docking principle that docks query compound CTL to the active site of the target protein database [19]. Reverse virtual screening task was submitted, and top 900 results were obtained (Figure 3A) based on scpdb database, which was dedicated to pharmacophore screening [20]. Based on targets docking score and targets information searched from the UniProt database, the top ranked cytoskeletal proteins, chlorophyll proteins, cytochrome proteins, and other proteins with unclear functions were excluded. Then, cyclin-dependent kinase 2 (CDK2) was identified as a crucial cellular target of CTL. The conformation of docked compound was shown by PyMoL. The binding mode of CDK2 with CTL was analyzed and shown as three-dimensional structure (Figure 3B) and electrostatic surface combination (Figure 3C). The results showed that CTL forms hydrophobic interaction with A31, A144, V64, F80, and L134 residues around the cavity of CDK2, so that CTL was fixed inside of a CDK2 cavity. By analyzing the superposition of CDK2-CTL and CDK2-ATP structures, we found that CTL competitively occupied the active site of ATP binding CDK2 (Figure 3D). Thereafter, we investigated the interaction between CTL and CDK2 quantitatively by SPR technology. Figure 3E showed that CTL specifically bound to CDK2 as a concentration-dependent mode. The dissociation constant (KD) was 32.02 μM, demonstrating a strong binding between CTL and CDK2. In addition, we tested and verified drug target engagement in BV2 microglial cells using CETSA and DARTS assay. As shown in Figure 3F, CTL notably intensified the stability of CDK2 compared with control group in BV2 microglial cells. Moreover, CTL concentration-dependently suppressed protease induced-degradation of CDK2 in DARTS, confirming the direct interaction between CTL and CDK2 (Figure 3G).

### 2.4. Knockdown of CDK2 Antagonized CTL Effect on Neuroinflammation via AKT/IKKβ/NF-κB Pathway

**Figure 4:**
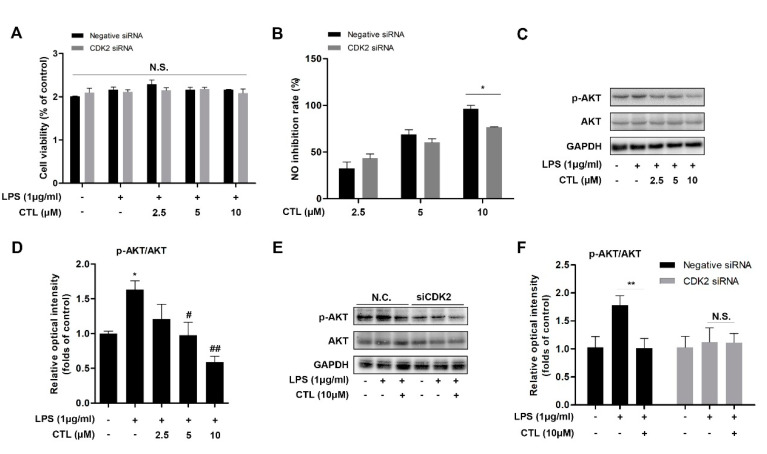
Knockdown of CDK2 antagonized CTL effect on neuroinflammation, CDK2 is necessary for CTL-mediated anti-inflammatory activity. (**A**) CDK2 siRNA-transfected BV2 microglial cells were treated with gradient concentrations of CTL and 1 μg/mL of LPS or vehicle for anti-inflammation assay. The cell viability was determined by MTT method. (**B**) The level of NO production was detected by Griess assay, and subsequently calculated its inhibition rate. *p* values were calculated by two-tailed *t*-test. * *p* < 0.05. (**C**) Phosphorylation and total expressions of AKT was determined by Western blot assay. (**D**) Quantitative analysis for relative phosphorylation levels of AKT. * *p* < 0.05 vs. control group. ^#^ *p* < 0.05, ^##^ *p* < 0.01 vs. LPS group. *p* values were calculated by ANOVA with Bonferroni’s post hoc test. (**E**) CTL inhibition on AKT phosphorylation was significantly reversed in CDK2 knockdown group compared with negative control group. (**F**) Quantitative analysis of negative control group and CDK2 siRNA group for relative phosphorylation levels of AKT. Data are expressed as mean ± SEM for three individual experiments. *p* values were calculated by two-tailed *t*-test respectively. ** *p* < 0.01. N.S., no statistical difference.

We explored the role of CTL-mediated anti-neuroinflammation effect in specific CDK2 gene silenced-BV2 microglial cells. We found that without affecting cell viability condition (Figure 4A), CTL-dependent inhibition on NO production was markedly reversed in CDK2 knockdown group (Figure 4B). AKT is a key upstream regulator of IKKβ/NF-κB pathway [21,22]. Here, we observed that LPS significantly induced AKT phosphorylation, which was suppressed by CTL as a concentration-dependent manner (Figure 4C,D). However, CTL inhibition on AKT phosphorylation was also significantly reversed in CDK2 knockdown group (Figure 4E, F). These results indicated that CTL attenuated AKT-dependent NF-κB signaling pathway activation by targeting CDK2. In sum, our results indicated that CTL-dependent neuroinflammation suppression was prominently reversed through CDK2 specifically knocking down, indicating that CDK2 as a key cellular target played a part in CTL-mediated anti-neuroinflammation.

## 3. Discussion

Accumulating evidences suggest that a crucial risk factor of neurodegenerative disorders is highly associated with neuroinflammation [23]. Microglial over-activation leads proinflammatory cytokines secretion, further triggering synaptic dysfunction and neuronal death, contributing to neuroinflammation-related pathological changes [24,25]. LPS stimulation can cause a profound immune response in the brain, which leads to the activation of microglia including BV2 [26]. A review reported that when CTL concentration is 0.8–2 μM, it has neuroprotective effect; when the concentration is in the range of 0.1–12.5 μM, CTL has anti-inflammatory activity; it can inhibit cell proliferation and mediate apoptosis when the concentration is around 10–100 μM [27]. In this work, we investigated anti-neuroinflammation role of natural sesquiterpene lactone CTL in LPS-induced BV2 microglia, and further explored its molecular mechanism. We confirmed CTL inhibited LPS-induced neuroinflammation response in BV2 microglia. CTL significantly inhibited the production of inflammatory mediators including nitric oxide, TNF-α, IL-6, and PGE2. Moreover, CTL down-regulated expressions of iNOS and COX2. Furthermore, CTL effectively inhibited AKT kinase phosphorylation, prevented phosphorylation and nuclear translocation of NF-κB p65 subunit, and attenuated LPS-induced NF-κB pathway activation.

Cellular target identification is a crucial prerequisite for clarifying biological mechanism of candidate drug. Reverse virtual screening which is based on “lock-key principle” of molecular docking is an effective method for cellular target identification [28]. The interaction between ligand and target protein is a process of molecular recognition, including electrostatic interaction, hydrogen bonding interaction, hydrophobic interaction, and van der Waals interaction. Through reverse virtual screening, the binding mode and affinity of ligand–target protein can be predicted. This method has been successfully used for cellular target identification and pharmacological mechanism researches of natural active molecules [29]. For example, reverse virtual docking was used to predict the targets of apple polyphenols in preventing colorectal cancer, and the main targets were guanosine 5′-monophosphate oxidoreductase (GMP reductase), guanosine triphosphatase (GTPase), and hypoxanthine-guanine phosphoribosyltransferase (HGPRT) [30]. Reverse virtual docking was also reported to predict the targets of an aminopyridine derivative as cyclin dependent kinases, Bcr-Abl kinase and KIT receptor kinase [31].

In this work, reverse virtual screening was performed to explore the cellular target of CTL. We identified that CDK2 was the target of CTL by screening scPDB protein structure library. CDK2 is a serine/threonine-protein kinase involved in DNA damage responds and cell cycle control [32,33,34]. Therefore, CDK2 is a promising target for neurodegenerative disorders, cerebral hypoxia, and cancers. Great progress has been made in the study of CDK2 in cell apoptosis, but its research in neuroinflammation needs to be further explored. Our molecular docking diagram and surface potential diagram showed that CTL bound to a cavity of CDK2 and formed hydrophobic interaction with alanine, valine, phenylalanine, and leucine residues around the cavity. Furthermore, the superposition analysis of CDK2-ATP and CDK2-CTL revealed that CTL occupied ATP binding site of CDK2. Therefore, we concluded that CTL affected CDK2 function by competitively binding with ATP to the active site of CDK2. On the other hand, SPR analysis suggested that CTL specifically bound to CDK2 with a dissociation constant at micromole level. Moreover, CETSA [35] and DARTS [36] are the important methods to verify the binding of small molecules to their target proteins. Our CETSA and DARTS analysis also confirmed that CTL could increase the resistance of CDK2 to high temperature and enzymes. Therefore, CDK2 was identified to be a potential therapeutic target for inhibiting neuroinflammation.

After cellular target identification, we tried to further elucidate potential anti-inflammatory mechanism. AKT kinase plays a key role in controlling neurogenesis including neuron location, dendritic development and synapse upstream formation [37,38]. NF-κB pathway is a canonical pathway in LPS-induced inflammatory response [18]. As a key upstream regulator of IKKβ/NF-κB pathway, AKT also plays an important role in regulating NF-κB-dependent inflammatory gene transcription [21,22]. Therefore, we tried to explore the relationship between CDK2, AKT, and NF-κB pathway in neuroinflammation. In this study, we identified an inhibitory role of CTL in regulation of LPS-activated AKT, IKKβ, IκB-α, and NF-κB p65 phosphorylation. Furthermore, we found that CTL significantly inhibited the activation of NF-κB-associated inflammatory gene transcription, indicating that CTL targets on CDK2 to affect AKT phosphorylation, thereby inhibiting NF-κB pathway activation to exert anti-neuroinflammation activity. Notably, we found that CDK2 knockdown significantly reduced the inhibitory ability of CTL on inflammatory mediators production. Moreover, CTL-dependent down-regulation on the phosphorylation of AKT, IKKβ, IκB-α, and NF-κB p65 was significantly reversed when CDK2 was silenced. This observation indicates that CDK2-mediated AKT/IKKβ/NF-κB pathway is highly involved in anti-neuroinflammation effect of CTL. Meanwhile, CDK2 may act as a vital cellular target responsible for anti-neuroinflammation therapy.

## 4. Materials and Methods

### 4.1. Chemical and Reagents

Costunolide (C15H20O2; molecular weight 232.32), abbreviated as CTL, with purity of 99.84% confirmed by MS and H1NMR data, was purchased from MedChemExpress LLC (Shanghai, China). LPS was from Sigma-Aldrich (St. Louis, MO, USA). Fatty acid-free bovine serum albumin (BSA) was obtained from Equitech-Bio (Kerrville, TX, USA). Antibodies against iNOS, COX2, GAPDH, p-AKT, AKT, p-IKKα/β, IKKβ, p-IκB-α, IκB-α, p-NF-κB p65, NF-κB p65, CDK2, Histone H3, and α-Tubulin were purchased from Cell Signaling Technology (Beverly, MA, USA). Recombinant human CDK2 protein was acquired from Sino Biological (Beijing, China).

### 4.2. Cells and Cell Culture Conditions

Murine BV2 microglial cell line was obtained from Peking Union Medical College, Cell Bank, Beijing, China. BV2 microglial cells were cultured under 5% CO_2_ and ambient O_2_ at 37 °C in the complete growth medium, including the high glucose Dulbecco’s Modified Eagle Medium (DMEM) containing 10% heat-inactivated fetal bovine serum (FBS, ABW, Uruguay), 100 IU/mL penicillin and 100 μg/mL streptomycin.

### 4.3. Cell Viability Assay

Cell viability assay was performed using the 3-(4,5-dimethyl thiazol-2-yl)-2, 5-diphenyl tetrazolium bromide (MTT) method. BV2 microglial cells were cultured in the complete growth medium containing CTL (0, 2.5, 5, 10, and 20 μM) and 1 μg/mL of LPS or vehicle for 24 h. Then, cells were processed by exchanging complete growth medium containing 0.5 mg/mL MTT (Sigma-Aldrich, St. Louis, MO, USA) and incubated at 37 °C in darkness for another 4 h. After removing the medium, formazan crystal products were dissolved in 100 μL dimethyl sulfoxide, and then the absorbance was detected at 570 nm.

### 4.4. Nitric Oxide Concentration Assay

BV2 microglial cells were cultured in complete growth medium containing CTL (0, 2.5, 5, and 10 μM) and 1 μg/mL of LPS or vehicle for 24 h, and subsequently 300 μL per well cell culture supernatants were collected. According to the manufacturer’s protocol, NO assay kit (Jiancheng, Nanjing, Jiangsu, China) was used for nitric oxide (NO) evaluation by Griess method.

### 4.5. Enzyme-Linked Immunosorbent Assay (ELISA) for TNF-α, IL-6 and PGE_2_

BV2 microglial cells were cultured in complete growth medium containing CTL (0, 2.5, 5, and 10 μM) and 1 μg/mL of LPS or vehicle. Culture supernatants were collected after 4 h and the level of TNF-α was determined with TNF-α ELISA kit (ExCell Bio, Shanghai, China). The level of IL-6 was determined with IL-6 ELISA kit (ExCell Bio, Shanghai, China) 8 h later. Moreover, BV2 microglial cells were treated for 24 h for PGE_2_ assay using ELISA kit from ENZO (ENZO, Farmingdale, NY, USA). TNF-α, IL-6, and PGE_2_ concentrations were measured at 450 nm according to the manufacturer’s protocol.

### 4.6. Western Blot Analysis

BV2 microglial cells were lysed in ice-cold RIPA buffer for half an hour to extract whole proteins. Nuclear and Cytoplasmic Protein Extraction Kit (NanJing KeyGen, Nanjing, Jiangsu, China) was used for nuclear and cytoplasmic proteins extraction. Enhanced BCA protein assay reagent (TransGen, Beijing, China) was used for determination of protein concentration. Ten microliters of protein of each group were separated by 10% SDS-PAGE and then transferred onto polyvinylidene fluoride (PVDF) membranes. After the membranes were blocked in 5% skim milk solution, the blots were incubated with diluted primary antibodies overnight at 4 °C. Then, anti-rabbit-IgG-HRP-conjugated or anti-mouse-IgG-HRP-conjugated antibodies were used as secondary antibodies. ECL signals were recorded by Tanon 5200 Imaging Analysis System (Tanon, Shanghai, China). Image J software was used for densitometry analysis of relative protein levels.

### 4.7. NF-κB Reporter Gene Luciferase Assay

Lipofectamine 2000 transfection reagent (Thermo, Waltham, MA, USA) and DNA were respectively diluted into equal amount of Opti-MEM medium (Thermo, Waltham, MA, USA). Diluted lipofectamine 2000 transfection reagent was mixed with DNA, and then incubated for 20 min at room temperature. After that, the complex was added to cells, and incubated for 6 h at 37 °C. Thus, BV2 microglial cells were co-transfected with 12 μg Renilla plasmids and 12 μg NF-κB plasmids transiently with lipofectamine 2000 transfection reagent (60 μL) for 48 h. BV2 microglial cells were subsequently treated with gradient concentration of CTL (0, 2.5, 5, and 10 μM) and 1 μg/mL of LPS or vehicle for overnight. Soon thereafter, cells were lysed, and dual luciferase reporter gene assay kit (Bioassaysys, Hayward, CA, USA) was used for detecting NF-κB reporter gene luciferase activity, and analyzed on a fluorescence spectrophotometer (PerkinElmer, Waltham, MA, USA).

### 4.8. Target Protein Identification for CTL

Reverse virtual screening was applied to identify the potential target of CTL. The basic principle was that binding strength was determined by the interaction energy (docking energy) of the ligand and the potential protein target. In order to predict the targets for a query ligand, structure databases containing numerous of protein targets was necessary under normal conditions. ScPDB protein structure library, a database dedicated to pharmacophore screening which including 4782 protein targets and 16,034 active sites was used in the present study. We used AutoDock software for docking and CTL was processed with ChemDraw to generate SDF file as the input ligand file. CTL was individually docked to each protein structure in scPDB database. A list of potential protein targets with docking energy score was screened and obtained. Top 900 were picked for further investigation. The main target protein for CTL was finally speculated based on the docking score list and its biological anti-inflammatory activity. CTL binding site in the target protein was shown by PyMoL and the interaction between CTL and target protein was analyzed.

### 4.9. Surface Plasmon Resonance (SPR) Assay

SPR analysis was performed on Biacore T200 system (GE Healthcare, USA). Carboxymethylated 5 (CM5) sensor chip surface was activated by 100 μL EDC mixed with 100 μL NHS. Flow rate was 10 μL/min for 10 min. Recombinant human CDK2 protein was diluted with acetic acid (pH 5.5) to 20 μg/mL, and then immobilized on the CM5 sensor chip using a standard amine coupling method. After blockage by ethanol amine (5 μL/min) for 10 min and solvent correction, gradient concentrations of CTL (12.5 to 200 μM) in the running buffer were injected as analytes. Kinetic parameters were computed with Biacore evaluation software (T200 Version 2.0).

### 4.10. Cellular Thermal Shift Assay (CETSA) [35]

BV2 microglial cells were treated with CTL (10 μM) for 2 h, while another group of cells were incubated for the same time duration with an equal amount of DMSO (0.1%) as control. Thereafter, cell pellets of two groups were harvested and resuspended with PBS respectively. Cell resuspension solutions were divided into 10 equal parts, and heated for 3 min individually at thermal gradient (40 to 58 °C), then cooling them at room temperature. The cells were collected and resuspended in KB buffer, then freeze-thawed repeatedly for 3–5 times using liquid nitrogen. The lysates were subsequently centrifuged, and the supernatants were analyzed by SDS-PAGE followed by immunoblotting for CDK2.

### 4.11. Drug Affinity Responsive Target Stability (DARTS) Assay [36]

BV2 microglial cells were lysed and diluted with 1× TNC buffer. The lysate was divided into five aliquots, and each aliquot was 50 μL. One aliquot was used as control (DMSO) group, while other aliquots were incubated with gradient concentration of CTL (0, 1, 10, and 100 μM) at room temperature for 1 h. Two microliters of diluted pronase solutions (2 μg/mL) was added respectively to the aliquots except control group at room temperature for further 15 min. At last, loading buffer was added to cease the reactions, and DARTS samples were tested by Western blot with the specific antibody of CDK2.

### 4.12. Transient Transfection with CDK2 siRNA

For siRNA knockdown, BV2 microglial cells were transfected with specific CDK2 or negative control siRNA using Lipofectamine RNAiMAX (Thermo, Waltham, MA, USA) in Opti-MEM (Thermo, Waltham, MA, USA). Specific CDK2 siRNA was designed and synthesized by GenePharma (Jiangsu, China). The nucleotide sequence of CDK2 siRNA was 5′-CGGAGCUUGUUAUCGCAAA-3′ (sense). The medium was refreshed with complete growth medium 6 h after transfection, and the following analysis were carried out after 48 to 72 h.

### 4.13. Statistics

All the data were analyzed and graphed with GraphPad Prism 6.0 software. The statistical analysis was done by one-way analysis of variance (ANOVA) and Dunnett’s post-hoc test. N.S. represents no significant differences, *p* < 0.05 was considered as statistically significant.

## 5. Conclusions

In summary, our work demonstrated the anti-neuroinflammation capability of CTL in LPS-induced BV2 microglia, and elaborated pharmacological mechanism by cellular target identification technique. These findings suggest CDK2 as a novel therapeutic target for neuroinflammatory diseases, and CTL may serve as crucial candidate compound for neuroinflammation-related diseases.
